# Integrated enzymes activity and transcriptome reveal the effect of exogenous melatonin on the strain degeneration of *Cordyceps militaris*

**DOI:** 10.3389/fmicb.2023.1112035

**Published:** 2023-04-06

**Authors:** Zhichao Zu, Siqi Wang, Yingming Zhao, Wenli Fan, Tianlai Li

**Affiliations:** ^1^Key Laboratory of Ministry of Education for Facility Horticulture, Shenyang, China; ^2^Key Laboratory of Protected Horticulture, National and Local Joint Engineering Research Center of Northern Horticultural Facilities Design and Application Technology, Shenyang, China; ^3^Liaoning Key Laboratory of Functional Cordyceps militaris, Shenyang, China; ^4^College of Horticulture, Shenyang Agricultural University, Shenyang, China; ^5^Liaoning Academy of Agricultural Sciences, Shenyang, China

**Keywords:** melatonin, *Cordyceps militaris*, strain degeneration, antioxidant system, cordycepin

## Abstract

As a valuable medicinal and edible fungus, *Cordyceps militaris* has been industrialized with broad development prospects. It contains a lot of bioactive compounds that are beneficial to our health. However, during artificial cultivation, strain degeneration is a challenge that inhibits the industrialization utility of *C. militaris*. Exogenous melatonin (MT) can scavenge for reactive oxygen species (ROS) in fungus and can alleviate strain degeneration. To establish the significance and molecular mechanisms of MT on strain degeneration, we investigated the third-generation strain (W5-3) of *C. militaris via* morphological, biochemical, and transcriptomic approaches under MT treatment. Morphological analyses revealed that colony angulation of *C. militaris* was significantly weakened, and the aerial hypha was reduced by 60 μmol L^–1^ MT treatment. Biochemical analyses showed low levels of ROS and malondialdehyde (MDA), as well as increasing endogenous MT levels as exogenous MT increased. RNA-Seq revealed that compared with the control, several antioxidant enzyme-related genes were up-regulated under 60 μmol L^–1^ MT treatment. Among them, *glutathione s-transferase* genes were up-regulated by a factor of 11.04. In addition, genes that are potentially involved in cordycepin, adenosine and active compound biosynthesis for the growth and development of mycelium were up-regulated. Collectively, these findings provide the basis for further elucidation of the molecular mechanisms involved in *C. militaris* strain degeneration.

## 1. Introduction

*Cordyceps militaris* is a valuable medicinal and edible fungus that has been intensively factory-produced. Despite the increased recognition of its significance, strain degeneration during the cultivation process of *C. militaris* leads to cyst deformation, reduction of the number of fruiting bodies, the content of nutrients and main active ingredients, or in severe cases, the failure to produce cysts (Das et al., 2010; Lin et al., 2010; Liu et al., 2020).

Strain degeneration is a major challenge in stable industrial development of *C. militaris*. Previous studies on overexpression of antioxidant enzyme genes in degenerated strains revealed that mutant strains were more capable of scavenging for intracellular reactive oxygen species (ROS). Moreover, their antioxidant enzyme activities were accordingly increased and grew substrates, suggesting that elevated intracellular ROS levels in strains led to *C. militaris* degeneration (Xiong et al., 2011). In our previous study, we found that morphologies of the colonies, mycelium, and spores of the third generation of successive cultures of *C. militaris* strain W5 began to change, with irregular rhythmic rings, gradual lightening in color, and the surface of mycelium begun to constrict and depress (Jiang et al., 2022). The degenerated strains exhibited significantly elevated ROS levels at mycelial tips and reduced antioxidant enzyme activities in the Ascorbate-Glutathione (ASA-GSH) cycle, confirming that W5 degenerated from the third generation and that ROS accumulation was a key factor in its degeneration (Yu, 2020). Yin (2018) subcultured *C. militaris* YCC for six consecutive generations. Findings from transcriptome sequencing of each generation of mycelia suggested that strain degeneration may be associated with toxin biosynthesis, energy metabolism, DNA methylation and chromosomal reconstruction.

In plants, melatonin (MT) is an indole-like tryptamine, plays important roles in accelerating seed germination, promoting growth and development, and enhancing resistance to abiotic stressors (Stürtz et al., 2011), such as drought (Zhao et al., 2021), salinity (Liu et al., 2021), high temperature (Qi et al., 2021), and nutrient deficiency (Li, 2019). As a multifunctional molecule, MT has the potential to improve plant stress resistance by enhancing the scavenging of ROS, thereby protecting plants from the adverse effects of abiotic stress (Fan et al., 2022). Studies have reported on MT treatment-induced resistance to stress in *Lentinus edodes*, which reduces active oxygen metabolism in cells (Jia, 2021). Moreover, MT has hydrophilic and lipophilic properties, and can enter the nucleus through the cytoplasm to play its role in intracellular free radical scavenging by increasing antioxidant enzyme activities, regulating the ASA-GSH cycle (Zheng et al., 2019), and participating in cellular pathways (Arnao and Hernández-Ruiz, 2021) to effectively scavenge for excess hydroxyl ions, hydrogen peroxide, superoxide negative ions, and other free radicals (He, 2021; Jia et al., 2021).

In this study, *C. militaris* W5-3 was used as the material in investigating the molecular regulatory mechanisms of MT in alleviating the degeneration of *C. militaris* initial strain. Our findings provide a theoretical basis and technical support for stable production of *C. militari*s.

## 2. Materials and methods

### 2.1. Materials and treatment

The *C. militaris* strain (W5) was conserved in the edible mushroom laboratory of Shenyang Agricultural University, while the third generation of early degenerate strain (W5-3) was used in succession cultures. About 0.018 g of MT (Solarbio, China) was weighed and dissolved in 1 ml of 70% ethanol that was mixed with ultra-pure water to form a concentration gradient of 0.5 μmol L^–1^ (M0.5), 1 μmol L^–1^ (M1), 10 μmol L^–1^ (M10), 20 μmol L^–1^ (M20), 60 μmol L^–1^ (M60), 100 μmol L^–1^ (M100), 150 μmol L^–1^ (M150), 250 μmol L^–1^ (M250), and 300 μmol L^–1^ (M300). Then, the same volume of different concentrations of MT were added to the PDA medium (Liu et al., 2008).

Using disposable inoculation needles, we spotted the W5-3 strains onto the center of PDA medium dishes with different MT concentrations. Then, they were incubated in a 22°C, 65% humid environment under a 4/20 h day/night cycle. Each treatment contained eight replicates.

### 2.2. Color change and ROS distribution analysis

Growth rates of the mycelium were recorded at 3 and 16 days using the crossover method. After 16 days of incubation, morphological characteristics and color changes of the colonies were observed and imaged. Color differences were detected using a CR-400 colorimeter with a yellow-orange color sample with *L*, *a*, and *b* values of 72, 20, and 62, respectively. The growth of strains at 15 days of incubation was investigated, and the average daily growth was calculated with six replicates for each treatment. The W5-3 mycelium was stained with the DCFH-DA ROS fluorescent probe (Solarbio, China), incubated in the dark for 25 min, rinsed with NaCl solution (serum-free solution is sufficient) and the ROS in mycelial cells observed using a laser confocal microscope (Zhao et al., 2014).

### 2.3. Analysis of superoxide anion and hydrogen peroxide levels

Malondialdehyde (MDA) and ROS levels were determined using the thiobarbituric acid (TBA) method (Zhao et al., 1994). The hydrogen peroxide (H_2_O_2_) and superoxide anion (O_2_*^–^*) kits (Solarbio, China) were used to assess H_2_O_2_ and O_2_*^–^* levels, respectively, as previously reported (Li et al., 2017). Assays were performed in triplicates.

### 2.4. Analysis of endogenous MT

High-performance liquid chromatography was used to assess MT levels. The mobile phase was acetonitrile: 50 mM Na_2_HPO_4_/H_3_PO_4_, pH = 4.5 (15:85), the mobile phase flow rate was 1.0 ml min^–1^, and the injection volume was 10 μl. The fluorescence detector was used for measurement, with emission and excitation wavelengths of 348 and 280 nm, respectively. Three parallel experiments were performed for each sample.

### 2.5. Analysis of antioxidant enzymes

Glutathione reductase (GR) and ascorbic acid peroxidase (APX) activities were determined as previously described (Zhang et al., 2015); monodehydroascorbate reductase (MDHAR) activities were determined using the method described by Yang (2017); dehydroascorbic acid reductase (DHAR) activities were determined using the method of Qin (2009); ascorbic acid (ASA) activities were determined using the method of Zhang et al. (2015). ASA and glutathione (GSH) were measured using commercially procured kits (Solarbio, China). The assays were performed in triplicates.

### 2.6. Determination of cordycepin and adenosine content

Cordycepin and adenosine levels were determined *via* high-performance liquid chromatography (HPLC), as described by Huang et al. (2015). A C18 reversed-phase column (250 mm × 4.6 mm, 5 μm) was used for analyses; the mobile phase was water: methanol (85:15, V/V); the maximum flow rate was 1.0 ml min^–1^; the injection volume was 20 μl; the column temperature was 30°C; the detection wavelength was 260 nm; and the analysis was performed using a TCS SP8 liquid chromatograph.

### 2.7. RNA extraction and transcriptome analysis

Samples were extracted by scraping the mycelium from the PDA medium and sequenced by Nanjing Jisi Huiyuan Biotechnology Co. Ltd. Total RNA was extracted using the TRIzol reagent (Thermo Fisher, China), according to the manufacturer’s instructions. Extracted RNA was digested using RNase-free DNase to eliminate genomic DNA contamination. A Nanodrop was used to detect purity (OD260/280), concentration and nucleic acid absorption peak of RNA. Detection of RNA integrity was performed using Agilent 2100 (Agilent, America). The mRNAs were extracted using Poly(A) beads, fragmented and reverse-transcribed to cDNA, followed by high-throughput sequencing (Pertea et al., 2016). After determining that the sample was qualified, a library was constructed. The eukaryotic mRNA was enriched using magnetic beads with Oligo (dT), after which it was supplemented with a fragmentation buffer to randomly interrupt the mRNA. Using mRNA as the template, the first cDNA strand was synthesized with six base random primers. Then, the second cDNA strand was synthesized by adding the buffer, dNTPs, RNase H and DNA polymerase I. The cDNA was purified using AMPure XP beans. The purified double stranded cDNA was repaired at the end, supplemented with A tail and connected with the sequencing connector. Then, AMPure XP beads were used to select the fragment sizes. The Illumina HiSeq 2500 sequencing platform was used for library generation and sequencing while Fastp (v0.20.0) was used to control the quality of the original data. The number of unknown bases *N* < 5, sequences with 50% of the length of reads and base quality value <5 as well as joint sequences, were removed (Pertea et al., 2016).

Clean reads for each sample were sequenced against the reference Chrysalis genome (Benefit from Jilin Agricultural University). Reference genome alignment was performed using HISAT2 (v2.1.0) (Kim et al., 2019) after which all genes were functionally annotated, including comparisons with NR (Deng et al., 2006), Swiss-Prot (Apweiler et al., 2004), KEGG (Finn et al., 2013), COG (Tatusov et al., 2000), KOG (Koonin et al., 2004), GO (Ashburner et al., 2000), and Pfam databases by BLASN using the e value cutoff of 1e−5. Based on the results, the libraries were evaluated, gene expression analyses were performed, and differentially expressed genes were identified. Prior to differential gene expression analyses, for each sequenced library, read counts were adjusted using the edger program package through the one scaling normalized factor. The *p*-value was adjusted using *q* value. FDR <0.01 and |log2(fold change)| ≥ 1 was set as the threshold for significant differential expressions (Wang et al., 2010).

### 2.8. Analysis of gene expression

The RNA extraction was performed using RNeasy Plant Mini kit, as instructed by the manufacturer. The amount of RNA used was determined based on RNA concentration. The Prime Script TM II 1st Strand cDNA Synthesis Kit (Takara, 6210A, China) was used for cDNA synthesis and diluted 20 times. Real-time fluorescent quantitative PCR was performed according to instructions of 2 × SYBR qPCR (Takara, RR350Q, China). Since changes in its expressions are minimal and are almost constant under different conditions, GAPDH (KC683907) was used as the internal reference gene. The PCR assay was conducted in a 20 μl volume containing 2 μl diluted cDNA, 0.4 μl forward primer, 0.4 μl reverse primer, and 10 μl 2 × SYBR qPCR Master Mix under the following conditions: 95°C for 1 min, 39 cycles of 95°C for 15 s and, 60°C for 60 s. The primer sequences that were used in this study are shown in Supplementary Table 1. The results were calculated using the 2^–ΔΔct^ algorithm (Livak and Schmittgen, 2001). The primers used for PCR are listed in Table 1. The actin gene was the internal control.

**TABLE 1:** Primer sequence of target genes for RT-qPCR analysis.

Gene	Forward primer (5′-3′)	Reverse primer (5′-3′)
*GADPH*	GCAACGATCTCGTCGTCAA	TGATGTTCTGAGCGGCAC
*E2.4.1.34*	CTCATCACCTGCCACGTCCT	TCTTGCCCTCGCCAAAGTAC
*PGC1*	CTTTGAGCGAGATGTTCAGGAG	GAGATGCGACGAGTGTGTAATAAT
*GSR*	ACGTACCCTCGGTCGTGTTC	TGCTGCGTAAATGTGTGGTTGT
*GCLC*	TCCTGTTTTCCATCCCGAGT	TGTGAGAGTGATGGGGTATTCC
*GCLC*	AAATCACCGTCAAGCTCTTCTTC	ACGTGCCCTCAAACGACATG
*GST*	TACGGTCAGGGTGTCTGGTTC	GGGTACTGTTTCTCAGCGTCATAG
*MGST1*	TTCACCGAGAACCTAACTCCTTT	AGTATATGCGGCAATACCCTTG
*GST*	GACCCTGGACAGGCACTACG	CACGGCTCCTTTTGCTCATT
*ADSS*	CTGAGACGGGCGACTTGATT	TGGGGACGTTGAGGAACTTT
*RRM2*	TTCTTCCTGAAATTCCAAATGC	TCTCGGTGATCGTGTCTGTCTT
*CECR1*	TGCGGAGCAACCAACTATACC	GACTCTTCGCCCACGAGATC

### 2.9. Data processing and statistical analysis

Data were analyzed using SPSS 22.0 (SPSS Inc., Chicago, IL, USA) (to produce tables) and Origin 18.0 (64 Bit) (to produce plots) for the new complex polar difference significance analysis. *p* ≤ 0.05 was the threshold for statistical significance.

## 3. Results

### 3.1. Effects of MT on colony morphology and *C. militaris* mycelial growth

**FIGURE 1:**
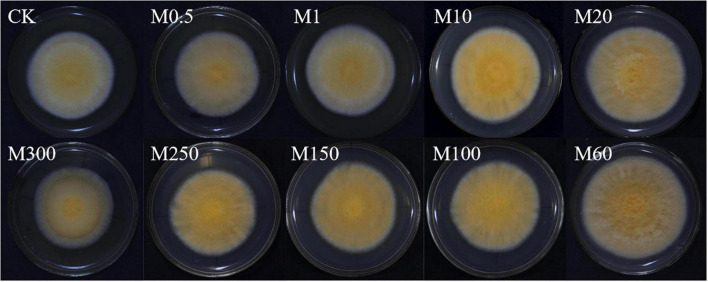
Effects of MT on colony morphology and *C. militaris* mycelial growth. Bar, 90 mm. The experiment was repeated ten times. A representative picture is shown here. M0.5, add 0.5 μmol L^–1^ of MT; M1, add 1 μmol L^–1^ of MT; M10, add 10 μmol L^–1^ of MT; M20, add 20 μmol L^–1^ of MT; M60, add 60 μmol L^–1^ of MT; M100, add 100 μmol L^–1^ of MT; M150, add 150 μmol L^–1^ of MT; M250, add 250 μmol L^–1^ of MT; M300, add 300 μmol L^–1^ of MT.

To determine the effects of MT on colony morphology and mycelium growth of *C. militaris*, we performed colony color observations and comparisons of W5-3 colony color changes treated with different MT concentrations. In Figure 1, it is shown that compared with the control, with increasing MT concentrations, the colony first became larger and then smaller, while the color changed from light to dark and then light. The colony of the M60 treatment was orange felt, the rhythm ring was significantly larger, the angle change was reduced, and the aerial mycelia was significantly reduced. Compared with CK, the growth rate was increased by 20.51%, and colony color Δ*L*, Δ*a*, Δ*b*, and Δ*E* values were significantly higher than those of other controls (Table 2). These findings imply that: M0.5, M1, and M300 treatments had no effects on mycelial growth and development while M10–M250 treatments promoted *C. militaris* colony morphology formation and mycelial growth, with the M60 treatment exhibiting the most significant effects.

**TABLE 2:** Comparison of color changes of W5-3 colonies treated with different concentrations of MT.

Treatment	Δ*L*	Δ*a*	Δ*b*	Δ*E*	Growth rate/mm d^–1^
CK	1.56 ± 0.074f	9.65 ± 0.22e	13.04 ± 0.24f	16.31 ± 0.04g	1.9069 ± 0.04fg
M0.5	1.95 ± 0.060e	9.70 ± 0.16e	12.74 ± 0.27f	16.13 ± 0.26g	1.9225 ± 0.01f
M1	2.13 ± 0.338e	9.71 ± 0.04e	12.49 ± 0.08f	15.97 ± 0.09g	1.941 ± 0.01ef
M10	3.48 ± 0.327c	11.17 ± 0.14d	20.84 ± 0.09d	23.91 ± 0.07d	2.0462 ± 0.01cd
M20	4.40 ± 0.161b	12.56 ± 0.03b	22.39 ± 0.12b	26.10 ± 0.07b	2.1688 ± 0.01b
M60	5.51 ± 0.090a	13.30 ± 0.13a	23.601 ± 0.20a	27.65 ± 0.21a	2.2972 ± 0.03a
M100	3.61 ± 0.261c	11.66 ± 0.06c	21.40 ± 0.32c	24.93 ± 0.03c	2.1054 ± 0.03c
M150	2.81 ± 0.048d	11.29 ± 0.1cd	20.43 ± 0.03de	23.51 ± 0.07e	2.0812 ± 0.00c
M250	2.02 ± 0.081e	9.74 ± 0.11e	20.13 ± 0.02e	22.456 ± 0.06f	2.0003 ± 0.012de
M300	0.10 ± 0.212ef	9.62 ± 0.21e	13.00 ± 0.23f	16.21 ± 0.07g	1.854 ± 0.018g

Δ*L* indicates luminance; Δ*a* indicates red; Δ*b* indicates yellow; Δ*E* indicates total chromatic aberration. Data are represented as means ± SD, *n* = 3. The different letters represent significant differences among treatments according to Duncan’s multiple range tests (*p* < 0.05). M0.5, add 0.5 μmol L^–1^ of MT; M1, add 1 μmol L^–1^ of MT; M10, add 10 μmol L^–1^ of MT; M20, add 20 μmol L^–1^ of MT; M60, add 60 μmol L^–1^ of MT; M100, add 100 μmol L^–1^ of MT; M150, add 150 μ mol L^–1^ of MT; M250, add 250 μmol L^–1^ of MT; M300, add 300 μmol L^–1^ of MT.

### 3.2. Effects of MT on ROS stain, O_2_^–^, H_2_O_2_, and MDA levels in *C. militaris*

**FIGURE 2:**
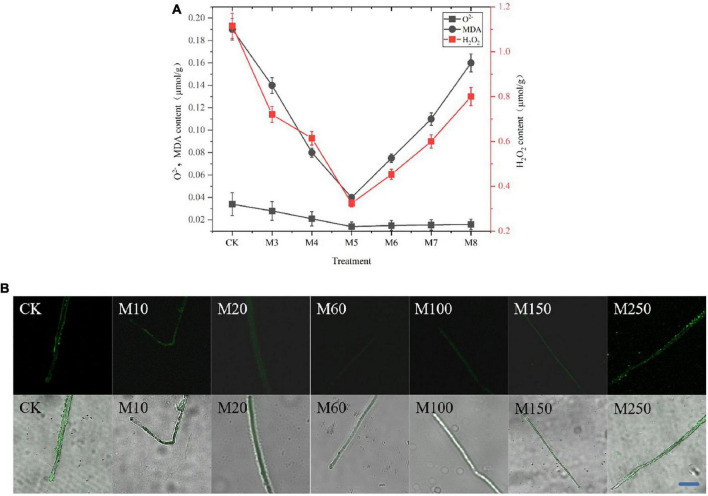
Effects of MT on ROS, O_2_*^–^*, H_2_O_2_, and MDA levels in *C. militaris.* **(A)** O_2_*^–^*, H_2_O_2_, and MDA content. Bars indicate mean ± SD (*n* = 3). The different letters represent significant differences among treatments according to Duncan’s multiple range tests (*p* < 0.05), the same letters represent no significant differences among treatments. Data are represented as means ± SD, *n* = 3. The experiment was performed three times with similar results. **(B)** Observe the ROS in the mycelia of *C. militaris* after MT treatment by laser confocal microscope with DCFH-DA ROS fluorescence probe. Bar, 10 μm. M10, add 10 μmol L^–1^ of MT; M20, add 20 μmol L^–1^ of MT; M60, add 60 μmol L^–1^ of MT; M100, add 100 μmol L^–1^ of MT; M150, add 150 μmol L^–1^ of MT; M250, add 250 μmol L^–1^ of MT.

To determine the scavenging capacity of MT, we measured the O_2_*^–^*, H_2_O_2_ as well as MDA levels and observed the ROS in the mycelia of *C. militaris*. As shown in Figure 2A, the ability of exogenous MT treatment to inhibit ROS production exhibited an increasing and then decreasing trend, and was always higher than the control. The O_2_*^–^* and H_2_O_2_ levels in the M60 treatment group were the lowest, decreasing by 60.29 and 66.73%, respectively, while the MDA levels decreased by 75.51%, when compared with CK. Moreover, in Figure 2B, the M60 fluorescence intensity was the weakest, indicating the lowest content of ROS in the mycelium and the strongest ability to inhibit ROS production.

### 3.3. Effects of exogenous MT on endogenous MT levels in *C. militaris*

**FIGURE 3:**
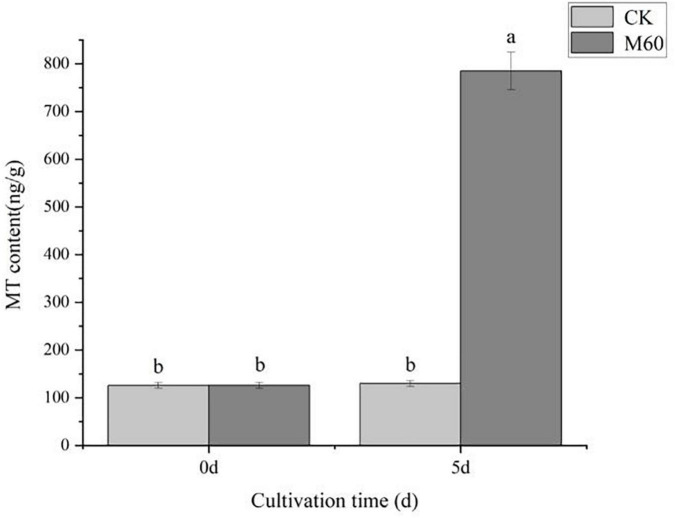
Effects of exogenous MT on endogenous MT levels in *C. militaris.* The different letters represent significant differences among treatments according to Duncan’s multiple range tests (*p* < 0.05). Data are represented as means ± SD, *n* = 3. M60, add 60 μmol L^–1^ of MT.

In plants, endogenous MT is involved in a variety of cellular activities as an important antioxidant, and the mechanism of endogenous MT in fungi to delay degeneration is still in its infancy. To speculate whether the action of endogenous MT in *C. militaris* is influenced by exogenous MT, we measured MT levels before and after MT application. The initial endogenous MT levels in *C. militaris* were 126 ng g^–1^. On day 5, the endogenous MT levels of mycelia were measured. In Figure 3, it was established that M60 treatment, namely, exogenous addition of 60 μmol L^–1^ MT, MT content increased by 523.02% compared to 5 days ago, significantly increased endogenous MT levels. We can thus speculate that the addition of MT in *C. militaris* can induce an increase in endogenous MT levels, thus acting to alleviate the degradation of the strain.

### 3.4. Differentially expressed genes in *C. militaris*

**FIGURE 4:**
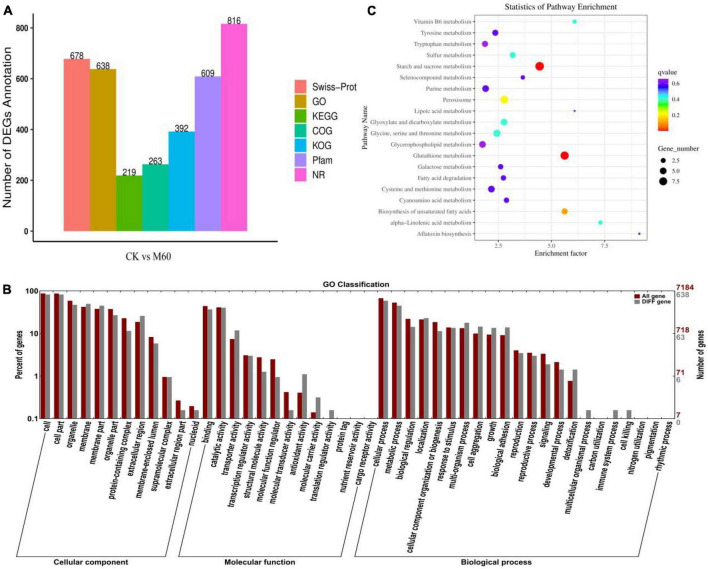
Statistics and analysis of DEGs in *C. militaris* strains. **(A)** Number of genes annotated in Swiss-Prot, GO, KEGG, COG, KOG, Pfam, and NR. **(B)** Go enrichment analysis in CK vs. M60. The abscissa is the GO classification, the left side of the ordinate is the percentage of the number of genes, and the right side is the number of genes. This figure shows the gene enrichment of each secondary function of GO under the background of differentially expressed genes and all genes, reflecting the status of each secondary function under the two backgrounds. **(C)** KEGG enrichment analysis in CK vs. M60. Each row in the figure represents a KEGG path. The abscissa is the enrichment factor, indicating the ratio of the proportion of genes annotated to the pathway in the differential gene to the proportion of genes annotated to the pathway in all genes. The larger the enrichment factor, the more significant the enrichment level of differentially expressed genes in this pathway. The color of the dot represents *q* value, and the size of the dot represents the number of differential genes in the annotation pathway. M60, add 60 μmol L^–1^ of MT.

Combined with mycelial growth and ROS accumulation in *C. militaris* in each group after exogenous MT treatment, it was found that the M60 dose was the most favorable for growth and development of the strain. RNA-Seq revealed 40.43 Gb data (Table 3), of which all clean reads were 6.34 Gb, and the percentage of Q30 bases was 92.19% and above. These findings indicate that transcriptome sequencing in this study was high enough for subsequent analyses. Functional annotation was performed on all genes and referenced reference genomes, which revealed 9,124 gene annotation results. A total of 678, 638, 219, 263, 392, 609, and 816 genes were annotated in Swiss-Prot, GO, KEGG, COG, KOG, Pfam, and NR, respectively (Figure 4A). The screening criteria for differential genes were | log2FC| ≥ 1, FDR ≤0.05. A total of 844 DEGs were identified for comparison in CK vs. M60, including 535 up-regulated and 309 down-regulated genes (Supplementary Table 1).

**TABLE 3:** Transcriptome sequencing data of *C. militaris* under control and MT treatment.

Sample	ReadSum	BaseSum	GC (%)	*N* (%)	Q20 (%)	CycleQ20 (%)	Q30 (%)
CK1	24,431,748	7,329,524,400	57.50	0.00	97.03	100.00	92.19
CK2	21,714,840	6,514,452,000	57.81	0.00	97.05	100.00	92.24
CK3	23,517,421	7,055,226,300	57.90	0.00	97.01	100.00	92.28
MT1	22,344,698	6,703,409,400	58.20	0.00	97.14	100.00	92.45
MT2	21,146,382	6,343,914,600	58.06	0.00	97.14	100.00	92.46
MT3	21,619,430	6,485,829,000	58.20	0.00	97.02	100.00	92.28

GO enrichment analysis of DEGs in CK vs. M60 (Figure 4B) yielded 638 GO functional terms, and were grouped into three major categories of biological processes, cellular components, and molecular functions. A total of 47 subclasses were classified into three major categories. Many DEGs were enriched in cells and cellular components (521, 521), followed by cellular processes (373), membranes (315), organelles (299), metabolic processes (286), catalytic function processes (286), and catalytic activity (256). The enrichment in cells and cell components indicates that MT has a greater impact on *C. militaris* mycelial growth during the development process, while enrichments in metabolic processes and catalytic activities indicate that MT has a significant impact on metabolism of active oxygen and accumulation of active substances in *C. militaris* during the development process, consistent with previous research results (Jiang et al., 2022).

The DEGs in CK vs. M60 were compared with the KEGG database, and 219 DEGs were annotated. A total of 20 pathways were enriched (Figure 4C), in which starch and sucrose metabolism, glutathione metabolism, peroxisome, glycerol phospholipid metabolism, biosynthesis of unsaturated fatty acids, galactose metabolism, and fatty acid degeneration are associated with maintenance of normal biological functions in cells; purine metabolism is involved in regulating energy supply and metabolite synthesis; tyrosine metabolism, tryptophan metabolism, glycine serine and threonine metabolism, and cysteine and methionine metabolism are associated with improvement of stress resistance. We postulated that exogenous MT improves stress resistance and alleviates the damage caused by strain degeneration by regulating cell homeostasis and energy supply.

### 3.5. Effects of MT on mycelial cell development of *C. militaris*

**FIGURE 5:**
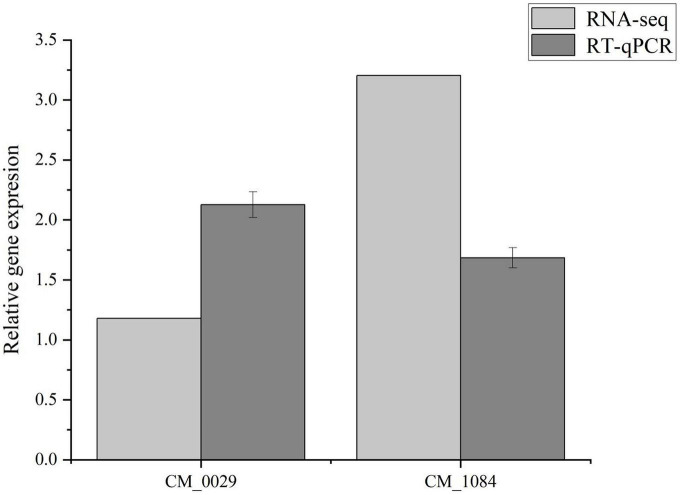
Validation of two DEGs in RNA-Seq using RT-qPCR. The different letters represent significant differences among treatments according to Duncan’s multiple range tests (*p* < 0.05), the same letters represent no significant differences among treatments. Data are represented as means ± SD, *n* = 3. The experiment was performed three times with similar results.

In the MAPK signaling pathway, *1,3-β-glucan synthase component* (CM_0029, Supplementary Table 1) and *Glycerophosphate diester phosphodiesterase* (CM_1084, Supplementary Table 1) in the lipid metabolism pathway are key enzymes for maintaining lipid remodeling and synthesis (Supplementary Table 1), playing an important role in cell wall and membrane structure synthesis (Lai, 2017). Elongation and growth of fungal cell walls require a balance between hydrolysis of existing cell walls and synthesis of new cell walls. β-Glucan is the main component in fungal cell walls. Expansion of the fungal cell wall includes polysaccharide synthesis and hydrolysis (Wessels, 1988). *1,3-β-glucan synthase* synthesizes chain-like β-1,3-glucan by using uridine diphosphate glucose (UDP-Glucose, UDPG) inside the cell membrane as a substrate and sending it into the cell wall space outside the membrane (Beauvais et al., 2001). β-Glucans hydrolyze the existing cell wall to allow the insertion of newly synthesized cell wall components without damaging the integrity of the cell wall (David, 2004). Similarly, *Glycerophosphate diester phosphodiesterase* is a key enzyme in the phospholipid metabolism pathway in organisms and plays an important role in maintaining the precursor triglyceride during lipid remodeling and synthesis. It hydrolyzes glycerophosphodiester substrates, lyses phospholipids, phospholipids, and nucleotide substrates to provide free phosphate to the body and maintain cellular homeostasis (Raetz, 1986; Jackson et al., 2007). After MT treatment, *1,3-β-glucan synthase component* gene (CM_0029, Supplementary Table 1) and *Glycerophosphate diester phosphodiesteras*e gene (CM_1084, Supplementary Table 1) were up-regulated, consistent with RT-PCR results (Figure 5), indicating that MT treatment had a significant impact on mycelial cell development.

### 3.6. Effects of MT on antioxidant capacity of *C. militaris*

**FIGURE 6:**
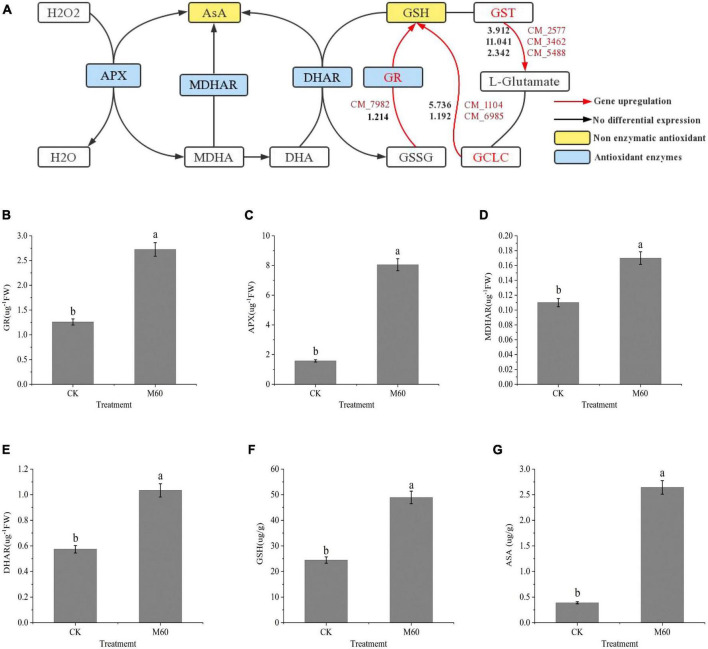
ASA-GSH cycle alleviates oxidative stress in *C. militaris* strain CK vs. M60 after MT treatment. **(A)** ASA-GSH cycle and glutathione metabolic pathway. The red mark next to the red line represents the DEGs, and the black number represents the DEGs expression. **(B)** GR content of CK and M60. **(C)** APX content of CK and M60. **(D)** MDAHR content of CK and M60. **(E)** DHAR content of CK and M60. **(F)** ASA content of CK and M60. **(G)** GSH content of CK and M60. The different letters represent significant differences among treatments according to Duncan’s multiple range tests (*p* < 0.05). Data are represented as means ± SD, *n* = 3. M60, add 60 μmol L^–1^ of MT. H_2_O_2_, hydrogen peroxide; ASA, ascorbic acid; GSH, glutathione; APX, ascorbic acid peroxidase; MDHAR, monodehydroascorbate reductase; DHAR, dehydroascorbic acid reductase; GR, glutathione reductase; MDHA, monodehydroascorbic acid; DHA, dehydroascorbic acid; GSSG, glutathione oxidized; GCLC, recombinant glutamate cysteine ligase; GST, glutathione S-transferase.

Excess ROS accumulation is associated with *C. militaris* degeneration. Its accumulation results in oxidative stress, which induces the onset of a series of transcriptional activities to alleviate oxidative stress. Physiologically, GSH is an important non-enzymatic antioxidant for scavenging ROS, and in the glutathione metabolic pathway (Figure 6A), glutamic acid cysteine synthetase (*GCL*) is an important rate-limiting enzyme for GSH synthesis. The *GCL* catalytic subunit (*GCLC*, CM_1104, CM_6985, Supplementary Table 1) plays a catalytic role in *GCL* and contains all the binding sites of GCL substrates as well as all catalytic subunits. Thus, it has all the catalytic activities of *GCL* and can be key in regulating GSH synthesis (Yan et al., 2013). The *GCLC* gene (CM_1104, CM_6985, Supplementary Table 1) was significantly up-regulated after MT treatment. In the pathway, GSSG was re-reduced to GSH by NADPH-supplied hydrogen under GR actions (CM_7982, Supplementary Table 1), and enhanced activities of GR promoted GSSG reduction to GSH, resulting in elevated GSH levels, while expressions of *Glutathione S-transferase* (GST, CM_2577, CM_3462, CM_5488, Supplementary Table 1) were up-regulated, which in turn promoted *L-glutamate* synthesis and catalyzed the activities of GCLC with *L-cysteine* to promote GSH synthesis. The GSH and ASA participate in the ASA-GSH cycle to scavenge for ROS under the synergistic effects of important antioxidant enzymes (GR, APX, MDHAR, and DHAR). In this study, levels of both non-enzymatic antioxidants and antioxidant enzymes were markedly increased after MT treatment (Figures 6B–G), while the levels of ASA and GSH in the mycelium increased by 104.97 and 680.47%, respectively, compared with CK. Findings from RT-PCR were consistent with the trend of gene expression in RNA-Seq, which proved that sequencing results had a high accuracy (Figure 5).

### 3.7. Effects of MT on accumulation of active components in *C. militaris*

**FIGURE 7:**
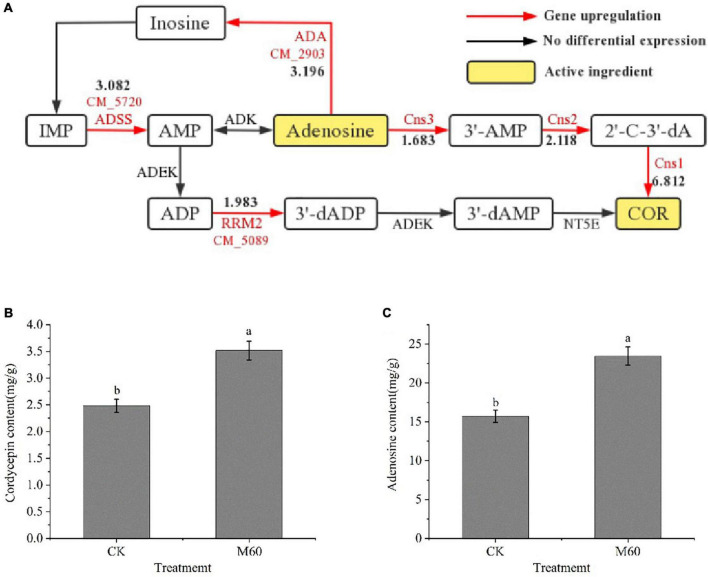
Accumulation of active components in *C. militaris* strain CK vs. M60 after MT treatment. **(A)** Biosynthetic pathway of cordycepin and expression of related DEGs. The red mark next to the red line represents the DEGs, and the black number represents the DEGs expression. **(B)** Cordycepin content of CK and M60. **(C)** Adenosine content of CK and M60. The different letters represent significant differences among treatments according to Duncan’s multiple range tests (*p* < 0.05). Data are represented as means ± SD, *n* = 3. M60, add 60 μmol L^–1^ of MT. IMP, inosine monophosphate; AMP, adenosine monophosphate; ADP, adenosine diphosphate; COR, cordycepin; ADSS, adenyl succinate synthetase; ADEK, adenylate kinase; NT5E, 5′-nucleotide.

Adenosine is the precursor of cordycepin (COR) synthesis, and cordycepin can be detoxified intracellularly by adenosine deaminase (ADA) to remove amino groups to produce *non-cytotoxic 3′-deoxyinosine* (Xia et al., 2017). Xia et al. (2017) predicted and verified the biosynthetic pathway of cordycepin in *C. militaris* (Figure 7A). First, inosine monophosphate (IMP) is generated by phosphorylation of adenosine monophosphate (AMP) under the actions of adenyl succinate synthetase (ADSS) and adenylate kinase (ADEK). Then, ADEK produces adenosine diphosphate (ADP) and 3′-dADP and 2′-dADP under the actions of *Ribonucleoside-diphosphate reductase* (*RRM2*.CM_5089, Supplementary Table 1) subunit, and 3′-dADP by the actions of ADEK. 3′-dAMP is generated by the actions of ADEK, and finally, 3′-deoxyadenosine, is generated by 5′-nucleotide (NT5E). In this study, gene expressions of *ADA* (CM_2903, Supplementary Table 1), *ADSS* (CM_5720, Supplementary Table 1) and *RRM2* (CM_5089, Supplementary Table 1) were up-regulated in purine metabolism. *Cns1-3* is a key gene in cordycepin biosynthesis and transformation (Xia et al., 2017). After MT treatment, levels of cordycepin and adenosine in mycelia were significantly increased (Figures 7B, C), and expressions of genes related to cordycepin synthesis were up-regulated, consistent with findings from RT-PCR.

## 4. Discussion

### 4.1. Exogenous MT promotes endogenous MT accumulation

Physiologically, MT plays an important role in antioxidant systems (Hernández-Ruiz et al., 2004). Supplementation of exogenous MT up-regulates the expressions of genes related to MT synthesis, including TDC, T5H, SNAT, and ASMT, thereby promoting endogenous MT levels (Liu et al., 2018; Shekari et al., 2021). In addition, endogenous MT biosynthesis is activated, and gene expressions of TDC, ASMT, and SNAT are significantly up-regulated with increasing endogenous MT levels (Wang et al., 2020). In previous studies, exogenous MT effectively promoted endogenous MT levels in bananas (Hu et al., 2017), grapes (Xu et al., 2017), and cherries (Wang et al., 2019). The increase in endogenous MT levels in fruits and vegetables may be related to delayed senescence and increased antioxidant activities (Wang et al., 2019). In this study, exogenous MT treatment significantly increased endogenous MT levels during storage. Increased MT levels enhance the stability of biological processes. Therefore, we postulated that exogenous MT promotes the accumulation of endogenous MT, regulates various metabolic pathways, and alleviates *C. militaris* degeneration.

### 4.2. MT treatment promotes the growth and development of *C. militaris* mycelium

During the subculture of *C. militaris*, thickness of cell walls of mycelial cells gradually decreases, and membrane structure gradually decreases to disappear (Jiang et al., 2022). During cell lipid remodeling and synthesis, *1,3-β-glucan synthase component* is an essential component specific to the fungal cell wall (Chen and Cui, 2007). *Glycerophosphoryl diester phosphodiesterase* is a key enzyme in phospholipid metabolism, catalyzing glycerophosphodiester hydrolysis in organisms to produce glycerol 3-phosphate and the corresponding small molecules, which is important for maintaining lipid synthesis in cells (Wang, 2011). In this study, expressions of *1,3-β-glucan synthase* and *Glycerophosphoryl diester phosphodiesterase* were up-regulated to different degrees, and after 60 μmol L^–1^ MT treatment, the mycelium was thicker and less divergent than before treatment. Constrictions and depressions on the mycelium surface were weakened, which led to the hypothesis that 60 μmol L^–1^ MT treatment affected the synthesis of key components for mycelial growth and development of *C. militaris* and weakened the strain effects. We postulated that 60 μmol L^–1^ MT treatment affected the synthesis of key components of mycelial growth and development, and reduced the effects of strain degeneration on mycelial growth and development.

### 4.3. MT treatment improves the antioxidant stress abilities of *C. militaris*

The balance of ROS metabolism in the body is a complex biological process. Degeneration of *C. militaris* strains results in ROS accumulation to cause oxidative stress, which initiates a series of transcriptional activities and gene expressions. As an antioxidant, MT can directly react with ROS to eliminate it and alleviate the oxidative stress-induced damage (Hardeland, 2010). Under low temperature stress, exogenous MT effectively reduced the accumulation of ROS and MDA in *Solanum melongena* L. seedlings (Wu et al., 2017). In *Arabidopsis* and *Cucumis sativus* L., the 100 μmol L^–1^ dose effectively removed excess ROS and MDA (Gao et al., 2014; Zhao, 2021). When leaves of *Kandelia candel* seedlings were treated with exogenous MT under low temperature stress, activities of SOD, GPX enzyme, and levels of antioxidant substrates (ASA and GSH) first exhibited increasing and decreasing trends, and the effects were significant when the MT dose was 50 μmol L^–1^ (Zheng et al., 2019). In fungi, low MT concentrations during *Saccharomyces cerevisiae* fermentation effectively reduced MDA and H_2_O_2_ levels and enhanced SOD and CAT enzyme activities, while high concentrations had inhibitory effects (Wang et al., 2015). In this study, we found that MT plays a significant role in regulating ASA-GSH, an important circulation system for scavenging ROS in *C. militaris*. The levels of ASA increased. It reacts with NADPH to reduce H_2_O_2_ to H_2_O, and at the same time, it is oxidized to form MDHA. One part of MDHA is reduced to ASA under the actions of MDHAR and participates in the ASA-GSH cycle again, while the other part of MDHA forms DHA through re-oxidation. In cells, DHA uses GSH as the substrate to regenerate ASA under the catalysis of DHAR, and the GSSG that is generated by this reaction is reduced to GSH by GR in the presence of NADPH (Zhang, 2007). After MT treatment, the levels of important antioxidant enzymes and non-enzyme antioxidants involved in the cycle and expressions of related genes were up-regulated, which indicated that 60 μmol L^–1^ MT played a very significant role in alleviating the strain degeneration for *C. militaris* by providing sufficient substrates for the ASA-GSH cycle, and accelerated the ROS removal efficiency to alleviate the ROS-induced damage in the strain.

### 4.4. MT treatment enhances the accumulation of active components in *C. militaris*

Previous studies found that exogenous MT effectively increased the soluble sugar and soluble protein contents in root systems of *Secale cereale* seeds under saline stress, differentially increased the levels of active components (ganoderic acid and astaxanthin), suppressed the intracellular ROS levels, and increased nitric oxide (NO) as well as salicylic acid (SA) levels in *Ganoderma lucidum* and *Platrotaenium maximum* (Reinsch.) Lund cells (Bao, 2016; Yue et al., 2019; Zuo et al., 2022). Fungal secondary metabolism is closely associated with oxidative stress, and successive cultures of fungi on artificial media often produce oxidative stress, which affects energy metabolism and subsequently results in changes in metabolites (Peng, 2014). Oxidative stress responses can be regulated *via* exogenous addition of non-enzymatic antioxidants. *C. militaris* degenerated during subculture. Adenosine levels in degenerated strains were lower than those of the normal strain, the ability to synthesize adenosine was weakened, and the cordycepin synthesis function was slowly lost (Yin, 2018). Exogenous GSH was found to be involved in elimination of intracellular ROS, promoted NADPH accumulation, changed the intracellular redox state, and improved the activities of the GPX, thereby promoting cordycepin synthesis (Li et al., 2021). Our findings are in tandem with those of previous reports, which found that exogenous MT altered the intracellular redox status, promoted cordycepin and adenosine levels, enhanced its own antioxidant capacities and slowed down the degeneration phenomenon. Moreover, MT significantly up-regulated the key genes regulating cordycepin synthesis in the purine metabolic pathway to promote cordycepin and adenosine synthesis. The MT concentration of 60 μmol L^–1^ significantly increased cordycepin and adenosine levels. This study is the first to report on the regulatory effects of exogenous MT on levels of active compounds in *C. militaris*.

## 5. Conclusion

In conclusion, exogenous MT is a simple and effective approach for alleviating strain degeneration and maintaining its stable production. Exogenous MT treatment of the *C. militaris* strain before cultivation can markedly reduce the negative effects of strain degeneration. *C. militaris* is a heterogeneous fungus in which genetic recombination frequently occurs. In previous experiments, it was found that the initial degenerated strains of *C. militaris* with appropriate amounts of MT were subjected to successive cultures, and the degenerative characteristics were still visible from the second generation, indicating that the ability of MT to alleviate the degeneration of the strains is time-sensitive. Therefore, supplementation of appropriate concentrations of MT at early stages of each *C. militaris* subculture can alleviate strain degeneration to a certain extent and maintain the good characteristics of the strain. Our findings form the basis for further studies on the roles of MT in alleviating the degeneration of *C. militaris*.

## Data availability statement

The datasets presented in this study can be found in online repositories. The names of the repository/repositories and accession number(s) can be found in this article/Supplementary material.

## Author contributions

ZZ, SW, and WF: methodology. ZZ, SW, and YZ: software. WF and TL: resources and funding acquisition. ZZ and WF: data curation. ZZ: writing—original draft preparation. All authors had read and agreed to the published version of the manuscript.
